# Application of Structural Equation Models for Elucidating the Ecological Drivers of *Anopheles sinensis* in the Three Gorges Reservoir

**DOI:** 10.1371/journal.pone.0068766

**Published:** 2013-07-30

**Authors:** Wang Duo-quan, Tang Lin-hua, Liu Heng-hui, Gu Zhen-cheng, Zheng Xiang

**Affiliations:** 1 National Institute of Parasitic Disease, Chinese Center for Disease Control and Prevention, WHO Collaborating Center for Malaria, Schistosomiasis and Filariasia, Shanghai, China; 2 Beijing Center for Disease Control and Prevention, Beijing, China; The George Washington University Medical Center, United States of America

## Abstract

**Objective:**

To identify the major ecological drivers for malaria vector density using the structural equation model (SEM) in the Three Gorges Reservoir.

**Method:**

An 11-year longitudinal surveillance of malaria vector as well as its related ecological factors was carried out in the Three Gorges Reservoir. The Delphi method was used to identify associated ecological factors. The structural equation model was repeatedly corrected and improved by the corrected index, combined with the actual situation. The final model was defined by relative simplicity, best fitting as well as the practicality.

**Result:**

The final model indicated that the direct effects of temperature, livestock, humidity, and breeding on the vector were 0.015, −0.228, 0.450, 0.516 respectively, their total effects on the vector were 0.359, −0.112, 0.850, and 0.043 through different pathways.

**Conclusion:**

SEM was effective and convenient in elucidating the mechanism by which malaria vector dynamics operated in this study. It identified that the breeding had the highest direct effect on vector and played a key role for mediating effect of temperature and humidity.

## Introduction

China Yangtze Three Gorges Project (TGP), as one of the biggest hydropower-complex projects in the world, is located at latitude 29°∼31°50′, longitude 106°20′∼110°30′, including 25 county-level divisions of Chongqing municipality and Hubei province and with the total population of 16 million [1]. The area around the Three Gorge Dam has a history of tertian malaria and subtertian malaria epidemic. There was no subtertian malaria after 1960, and the prevalence was controlled by the end of 1980s. No other malaria vectors except *An. sinensis* existed in the Three Gorges Reservoir based on the published literatures [2].

While the global and regional ecological change is often mediated through complex and large-scale processes, making the links between ecological change and disease difficult to demonstrate scientifically [3], there is a notable paucity of studies to confirm or refute whether, and how, ecological change plays this role. Many factors contribute to our poor understanding of the causes and mechanisms of disease emergence [4].

However, mathematical modeling with statistical analysis has played an important role in understanding the malaria transmission vectors and its epidemiology, and the most classical studies [5]–[7] have been based on the mosquito population vectorial capacity statics assumptions. Though an increasing number of empirical studies [8]–[9] indicating that the malaria vector distribution was related to ecological factors such as the natural, social as well as environmental variables, most of them [10]–[12] often separate problems into elements and then focus on the elements isolately, few studies have analyzed the underlying ecological drivers of the malaria vectors from the overall point of view. This may be due both to difficulty obtaining longitudinal parameter estimates and the limitations of traditional approaches to studying causal inference in complex systems [13].

A survey of current epidemiologic review [14]–[15] shows that most of the mathematical and quantitative work in epidemiology has resulted in what King and Soskoline have termed ‘associative models’. These are models that attempt to establish etiology by observing the associations of various risk factors with the occurrence of disease. However, the structural equation model (SEM) [16] is a modern powerful multivariate statistical method that allowing the evaluation of a series of variables. A distinct advantage [17] of SEM over conventional multiple regression analyses is that it has greater statistical power than does the latter, and its application in medicine improved more in-depth understanding of the relationship than the conventional epidemiologic methods. Although its application has been seen in many disciplines [18]–[21], it has yet to be extensively used in medical research and epidemiology [17]. Recently, concern [22] about the scarcity of SEM models in epidemiological research has been raised and urged epidemiologists to use SEM models more frequently. With its strength as a statistical tool to analyze complex relationships among variables, even posit and test causal relationships with no experimental data, and it allows researchers to explain the development of phenomena such as disease and health behaviors.

Nevertheless, the framework is a way of synthesizing and assessing multiple and complex sources of data and rigorously attribute a causal link which has great potential as a tool to guide current malaria elimination efforts. In this study, we will first decipher the complex relationship between the distribution of malaria vectors and its related ecological factors through the SEM. It is for the purposes of a sound understanding the more general behavior of malaria transmission and providing insight necessary to manage the disease by shaping and guiding vector control towards malaria elimination in China.

## Materials and Methods

### Study sites

**Figure 1:**
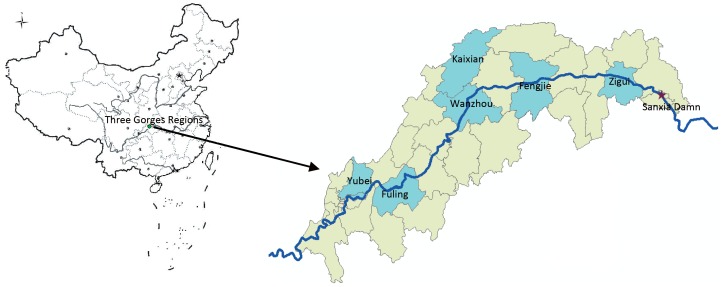
Location of study areas in Three Gorges Reservoir Area.

According to some socioeconomic factors and environmental features (e.g. paddy field, riparian zones) relating to the malaria vectors distribution, six natural villages (Zigui, Fengjie, Kaixian, Wanzhou,Yubei and Fuling) were selected from different zones in the Three Gorges Reservoir [23] (Figure 1). With informed consent and active cooperation of the villagers, this study was undertaken from 1997 to 2008 in the selected villages (population 500–1000 each). House design usually consisted of either one or two-room mud-daubed construction with a low, thatched roof. The eaves of most houses were open, which facilitated mosquito ingress and egress. The average family size was about five people per house, together with their chickens, often pigs. Cooking occurred typically inside the homes or under the eaves of a porch. Ten houses were selected upon receiving consent from household heads from each selected village when the survey was carried out [24].

### Mosquito collection

Mosquitoes were collected in each village every 15 days using electric motor mosquito catches (CN85202146) between May and October from 1997 to 2008. For each survey, mosquito's collections were carried out from 18:00 to 20:00 hour in the same 10 houses in the selected villages. The collected mosquitoes were taken to the laboratory and killed by suffocation with chloroform vapor. They were counted and identified morphologically using taxonomic keys [25], the density calculated as the number of female adults per house man hour.

### Ecological data collection

The Delphi method by expert opinion was used to identify ecological factors associated with the distribution of malaria vectors in this area. The result of positive coefficient, authority coefficient and coefficient of concordance in the evaluation system composed of twelve related index (Γ1: The average temperature of this month; Γ2: The average evaporation of this month; Γ3: The average temperature of last month; Γ4: The average number of pigs per house; Γ5: The average number of big livestock per house; Γ6: The average precipitation of this month; Γ7: The average precipitation of last month; Γ8: The average relative humidity of this month; Γ9: The average relative humidity of last month; Γ10: The annual average usage pesticides of per capita; Γ11: The annual average capita of paddy field; Γ12: The annual average capita of riparian area) for assessing the malaria vector distribution through 3 rounds of consultation of 26 advanced experts from over the country was 0.92, 0.76, 0.39 (χ^2^ = 38.84, n = 24, p<0.01) respectively, indicating that forecasting or assessing by the evaluation system should be adequate.

The value of related index on the basis of the evaluation system were collected from local department: the meteorological data from 1997 to 2008 were actual weather station records, and the data of monthly precipitation, temperature, relative humidity and evaporation for the period of this survey was provided by the Chongqing and Yichang branch of Chinese Meteorological Agency. The riparian area as well as paddy field area was gotten from the Bureau of the Land Resources and Houses Management in Yichang and Chongqing. The social economy of each village including the use of pesticides and number of livestock from 1997 to 2008 was provided by the Statistical Bureau in Chongqing and Yichang.

### Data Process

The ecological data (monthly average) was matched with the corresponding vector density (monthly average). The data distribution, correlation, regression as well as colinearity analysis was processed by the conventional statistical methods. All the analyses of this study were carried out using the SAS9.1.

### Exploratory Factor Analysis

By rotating the initial factor to establish the relationship between the latent factors and indicators using Varimax Law. An initial model evolved data fitting and the Delphi result had been put forward on the basis of the correlation and regression analysis as well as the exploratory factor analysis. The model was further screening based on the following aspects: 1) model fitting indicators within an acceptable range, 2) model fitting residual distribution reasonable, 3) correlation coefficient of equation in the model applicable, 4) parameter estimates of the model better than Delphi method.

### Confirmatory Factor Analysis

The exploratory factor analysis indicated that not only the coefficients between indicators and corresponding latent factor was statistically significant, but the correlation coefficients between ecological factors (F1, F2, F3, F4) and vector factor demonstrated a significant interrelationship. Thirteen observed indicators and forty four free parameters were analyzed in the priori model. According to the model t rule (t = p_ *_ (p+1)/2 = 13_ *_ 14/2 = 91), the parameters convergence was estimated using weighted least squares after 507 iterations.

### The Final Model

The final model was a modified, suitable standardized of applicable Delphi result, expert advisory as well as related literature review, the fit indices have been significantly improved compared to these initial models, standardized residual distribution was also smaller than the previous models.

## Results

No other malaria vectors except *An. sinensis* were caught during the surveillance.

### Exploratory Factor Analysis

Four latent factors as shown in Table 1 (Temperature, Livestock, Humidity, Breeding) were identified based on basic principles including scree plot, characteristic roots variance that is more than 1, common characteristic variance (70.5%) which is more than 70% of the total variance in combination with the Delphi result.

**Table 1:** The Varimax method rotated each indicator in load factor on the four potential factors.

Indicators	Factors	F1(Temperature)	F2(Livestock)	F3(Humidity)	F4(Breeding)
Γ1	The average temperature of this month	**0.8819**	0.0337	0.0865	−0.0105
Γ2	The average evaporation of this month	**0.7421**	−0.1909	−0.123	0.2881
Γ3	The average temperature of last month	**0.6754**	0.0236	0.0264	0.0291
Γ4	The average number of pigs per house	−0.0929	**0.9200**	−0.0959	−0.0818
Γ5	The average number of big livestock pigs per house	−0.0373	**0.9079**	−0.0244	0.0645
Γ6	The average precipitation of this month	−0.3719	−0.0453	**0.5684**	0.4222
Γ7	The average precipitation of last month	0.2439	0.0464	**0.755**	−0.0286
Γ8	The average relative humidity of this month	−0.5447	−0.1516	**0.6636**	−0.2134
Γ9	The average relative humidity of last month	0.0042	−0.1621	**0.7514**	−0.4151
Γ10	The annual average usage pesticides of per capita	−0.2604	0.3941	−0.0894	**−0.6676**
Γ11	The annual average capita of paddy field	0.212	0.6058	−0.0502	**0.6266**
Γ12	The annual average capita of riparian area	0.0312	0.1904	0.1411	**0.6688**

### Confirmatory Factor Analysis

**Figure 2:**
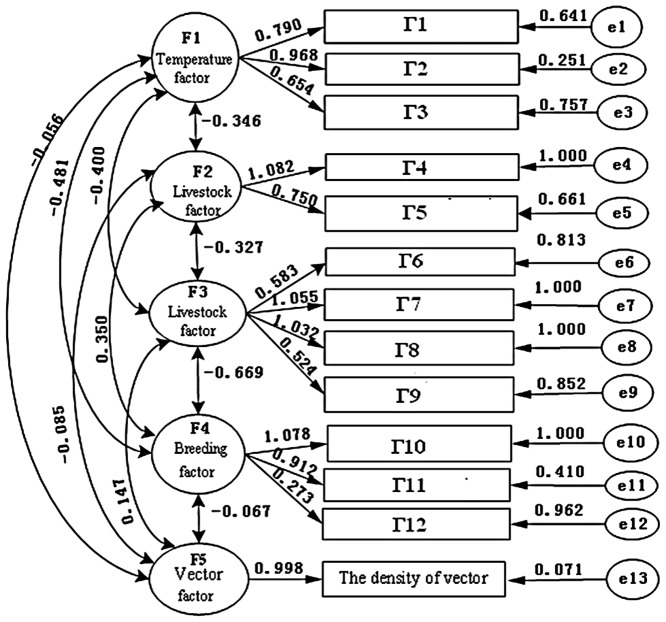
The initial model path diagram.

The initial model (Figure 2) indicated that the coefficients between the indicators and the corresponding latent factor was statistically significant (t>1.96, P<0.05), indicating that the observed indicators was dominated by each latent factor. The correlation coefficients between ecological factors (F1, F2, F3, F4) and the vector factor was −0.056, −0.085, 0.147, −0.067 (P<0.05), demonstrating a significant interrelationship between temperature, humidity, livestock, breeding and vector.

**Figure 3:**
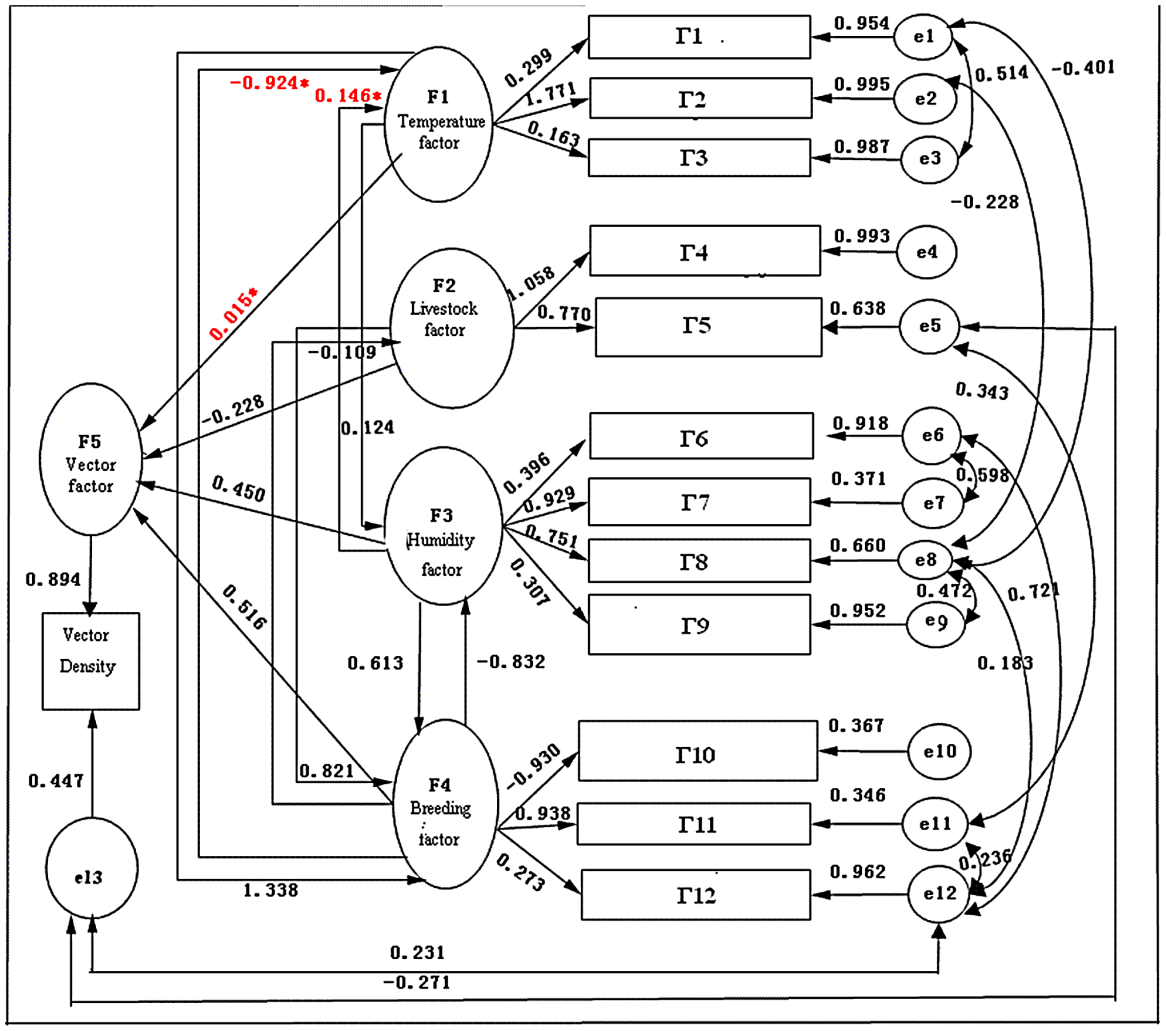
The final model path diagram.

On the other hand, the results also showed that most model fitting indicators were within the acceptable range except the fitted values of the model Ratio (CHI/df), residual standard root-mean-square (SRMR) and approximate root mean square error (RMSEA). The standardized parameter estimation in priori model (Figure 3) indicated that measurement coefficients were statistically significant (t>1.96, p<0.05). Most of fitting indexes reached a significant validity range. On the basis of the corrected model index (Modification Index, MI), combined with the Delphi result, the initial model was repeatedly corrected. Fitting process indicators showed that fitting indices like GFI(Goodness of Fit Index), AGFI(Adjusted Goodness of Fit Index), CFI(Comparative Fit Index), NFI(Normed Fit Index), NNFI(Non-normed fit index), AIC(Akaike Bayesian information criterion), CAIC (Akaike information criterion)and SBC (Schwarz Bayesian criterion) index gradually ameliorated, indicating that the degree of the data fitting of the model improved.

### The Final Model

The final model indicated that the fit indices have been significantly improved compared to those initial models (Table 2) and standardized residual distribution was also smaller than the previous models, 70% of the standardized residuals absolute value were less than 1.96, the mean of the residuals (1.53) and residual elements (1.79) were lower than the triangular average 1.96.

**Table 2:** Major fitting indexes of the final model.

Fitting index	x2/df	GFI	AGFI	RMSEA	CFI	NNFI	NFI	CN
Acceptable value	<5.0	>0.9	>0.9	<0.1	>0.9	>0.9	>0.9	>200
Fitting value	5.2200	0.9960	0.9987	0.1303	0.9961	0.9929	0.9952	207

The final model (Figure 3) indicated that the direct effects of temperature, livestock, humidity, and breeding on the vector were 0.015, −0.228, 0.450, and 0.516, while their indirect effects were 0.344, 0.116, 0.400, −0.473 respectively, and the total effects were 0.359, −0.112, 0.850, and 0.043, in turn (Table 3).

**Table 3:** The total effects of different potential factors on the vector factor in the final model.

Factor	Direct effect	Indirect effect	Total effect
F1→F5	0.015	0.344	0.359
F2→F5	−0.228	0.116	−0.112
F3→F5	0.450	0.400	0.850
F4→F5	0.516	−0.473	0.043

Since the breeding had the biggest (0.516) direct effects on the vector of all the ecological factors, and the temperature, the humidity as well as the livestock had an significant direct effects of 0.690, −0.307, and 0.316 on the breeding, so the breeding had played an important role in mediating the impact of these factors on the vector (Table 4), and the humidity demonstrated the highest total effect by the indirect effect of breeding, as well as the temperature exerted a total effect of 0.359 through the indirect interactions with humidity and breeding. However, the breeding had the smallest total effect by the indirect effect of livestock.

**Table 4:** Indirect effects of different potential factors on the vector factor in the final model.

Factor				Indirect	effects	From	different	pathways			
	F1	F2	F3	F4	F1→F3	F1→F4	F3→F1	F3→F4	F4→F2	F4→F3	Total
F1→F5	-	-	0.056	0.690	-	-	-	0.102	-	−0.504	0.344
F2→F5	-	-	0.424	−0.307	-	-	-	-	-	-	0.116
F3→F5	0.002	-	-	0.316	-	0.101	-	-	−0.019	0.400	0.400
F4→F5	−0.014	−0.031	−0.374	-	−0.052	-	−0.002	-	-	-	−0.473

## Discussion

### The first application of the model in this field confronted its challenge of refining the model towards more powerful and synthetic tool

Vector transmission system represent complex interactions and understanding the overall picture of the transmission chain require appropriate modeling methods of refining approaches towards a more powerful and synthetic structural model [26]. The performance of the structural equation models demonstrated in the recent social studies has proven beneficial in the exploration of novel ideas and approaches in epidemiology to understand complex interconnection, but its application to disease transmission has been very few. With the challenge of refining the model towards more powerful and synthetic tool, its application in disease systems, the first, perhaps is to elucidate the factors influencing malaria vector distribution. Here each factor was assembled in the overall model into the ecological picture of the transmission system.

### Compared with the conventional regression analyses, this model first revealed the complex causal mechanism for ecological drivers of *An. sinensis* from the overall and quantitative point of view reasonably as well as credibly

Compared to the most previous studies [11]–[13] using the traditional epidemiological method that explored their relationship between related factors and exposure isolately, the model based on the Delphi results has first gone beyond the traditional risk factor analysis used in public health for elucidating the underlying mechanism of ecological drivers for *An. sinensis* through the complex web of related factors that may ultimately impact the malaria vector distribution. The results from model fitting analysis showed that all the observational indicators with statistically significant regression coefficients could be explained by the latent factors. Most of the fitting indexes of the structural model fell within the acceptable range, and the final model showed reasonable standardized residual distribution. Further, the structural equations correlation coefficient corresponded to fieldwork reality as well as the Delphi result. Therefore, the structural model in this study was well reasonably established.

### The temperature exerted a significant effect on *An. sinensis* density mainly through the indirect effect of breeding

It is observed in numerous studies [27]–[30] that the temperature varied with season and such seasonal variations influence the density of malaria vector around the world. Further studies [29], [31]–[32] have indicated that temperature does not have a simple linear relationship with malaria vector density: within a certain range of temperature, malaria vector density increases with temperature, while extreme low and high temperature decreases the density. However, this SEM further elucidated the mechanisms by which temperature influence the malaria vector distribution through the breeding indirectly. This study indicated that although the temperature factor had a smaller and statistically insignificant direct effect on *An. sinensis*, it showed the largest indirect effect through breeding of all the factors. Accounting for its indirect effect, temperature exerted a high total effect on *An. sinensis* density. Therefore, this study further elucidated the mechanism behind temperature's impact on the distribution of *An. sinensis*.

### The humidity indicated the highest total effect on the *An. sinensis* of all through indirect effect from breeding in this region

Various studies [33]–[34] have suggested that increased humidity or rainfall was one of the primary causes of influencing the malaria vector density. As rainfall increases, malaria vector density can be impacted by the increase or decrease in breeding ground through bringing in more moisture or decrease it by stronger erosion [35]–[37]. However, Three Gorges Reservoir has a mild and humid climate suitable for the development of *An. sinensis* [38]. In this study, the humidity factor not only had a higher direct effect but also demonstrated the highest total effect of all factors on *An. sinensis* through the indirect effects of breeding. So, the humidity factor served as the primary driver for *An. sinensis* in the Three Gorges Reservoir.

### Unlike other ecological factors, the livestock had a negative effect on *An. sinensis* which suggesting some degrees of biological barrier effect

Similar to several studies [39]–[40] that showed a negative correlation between livestock and malaria vector, this study also found a negative effect from the livestock on *An. sinensis*. The final model indicated that one unit increase in livestock decreased 0.228 (directly) or 0.112 (indirectly) units of vector. Therefore, large domestic animals may prevent the human-anopheles contact, indicating some biological barrier effects in this region. This finding was closely related to the ecological behavior of *An. sinensis* which had a preference for animal blood and its accessibility to local hosts. It is noteworthy that the use of pesticides had a negative relationship with *An. sinensis* density (r = −0.1096) in this study, and Chen Huailu [41]–[42] et al. also found that the increase use of pesticides decreased *An. sinensis density* by 71.43% in similar region.

### Breeding exhibited the highest direct effect on vector and severed as a key role in mediating the effect of temperature and humidity

Numerous studies [43] have confirmed that the primary breeding ground of *An. sinensis* was rice fields as well as its related irrigation system in China, the survey by Chen Huailu et al. demonstrated that the rice fields constituted about 93% breeding ground of *An. sinensis* in this region, and the investigation from 2008 to 2009 also indicated that the density of *An. sinensis* was most correlated (RR = 11.84) to the rice field as well as the size of riparian area in the Three Gorge Reservoir [44]. Similarly, this study further unraveled the mechanism for the breeding through its highest direct impact (0.516) on vector as well as the key role in mediating the effect of temperature and humidity on vector.

Above all, this model has great potential as a pragmatic tool to help guide malaria elimination efforts and in particular to predict which are likely to be the most efficient and cost-effective strategies in different epidemiological settings. However, some potential limitations of this model should be mentioned. First, the goal of this model is not to prove causal relationships, but rather to collect sufficient evidence for reaching a verdict of causation which can justify or direct management action. Second, some poor or unreliable surveillance data may give misleading results. Third, socioeconomic factors such as the houses type, bet net-use should be supplemented for improving the model. Despite these limitations, this SEM is a powerful tool for exploring the potential impact of current and future interventions on malaria vector transmission in a systematic manner.
